# Incidence of trigger digits following carpal tunnel release: A nationwide, population-based retrospective cohort study

**DOI:** 10.1097/MD.0000000000007355

**Published:** 2017-07-07

**Authors:** Fu-Yu Lin, Oscar J. Manrique, Cheng-Li Lin, Hsu-Tang Cheng

**Affiliations:** a Department of Neurology; b Division of Plastic and Reconstructive Surgery, Department of Surgery, China Medical University Hospital, China Medical University School of Medicine, Taichung City, Taiwan; c Division of Plastic Surgery, Department of Surgery, Mayo Clinic, Rochester, MN, USA; d School of Medicine, Graduate Institute of Clinical Medical Science and School of Medicine, College of Medicine, China Medical University; e Management Office for Health Data, China Medical University Hospital, Taichung, Taiwan.

**Keywords:** carpal tunnel syndrome, trigger finger

## Abstract

The onset of trigger digits after carpal tunnel release (CTR) have been reported inconsistently across different studies. The aim of this study is to assess the incidence of trigger digits after CTR using nationwide population cohort data.

We conducted a retrospective cohort study using the Longitudinal Health Insurance Database 2000 (LHID2000) from the National Health Insurance Database (NHIRD) in Taiwan. The LHID2000 contained 1 million beneficiaries randomly selected from the year 2000 Registry for Beneficiaries in NHIRD. We identified 2605 carpal tunnel syndrome (CTS) patients received CTR from 2000 to 2010 (CTR cohort, n = 2605). For each CTR patient, 4 CTS patients without CTR were randomly selected in the control cohort from the general population frequency matched by age, sex, and diagnosed year (non-CTR cohort, n = 10,420). Both cohorts were followed up until the end of 2011 to investigate the occurrence of trigger digits. Adjusted hazard ratios (aHRs) with 95% confidence interval (CI) of trigger digits were estimated using the Cox proportional hazards model after controlling for age, sex, and comorbidities.

The CTR cohort had a mean follow-up period of 5.58 ± 3.18 years and the non-CTR cohort had a mean follow-up period of 5.90 ± 3.10 years. The overall risk of trigger digits was 3.63-fold greater in the CTR cohort than in the non-CTR cohort (incidence rate: 12.6 vs 3.38/1000 person-years, aHR: 3.63, 95% CI, 2.97–4.44). The incidence of postoperative trigger digits was highest in the 1st 6 months (incidence rate: 27.9/1000 person-years, aHR: 9.65, 95% CI, 5.27–17.7) and then significantly decreased over time.

CTR was significantly associated with the subsequent development of trigger digits, especially in the postoperative 6 months.

## 1 Introduction

Carpal tunnel is the passageway deep to the transverse carpal ligament between tubercles of the scaphoid and trapezoid bones on the radial side and the pisiform and hook of the hamate on the ulnar side, through which the tendons of the flexor digitorum profundus, the flexor digitorum superificialis, and the flexor pollicis longus pass.

The 2 most common nontraumatic hand disorders treated by plastic surgeons are carpal tunnel syndrome (CTS) and trigger digits. CTS occurs when the median nerve is compressed as it travels through the carpal tunnel, which is formed by the transverse carpal ligament (flexor retinaculum) superiorly and the carpal bones inferiorly. The hallmarks of CTS are pain, numbness, tingling, and muscle atrophy in the vicinity of the median nerve. Trigger digit results from a disparity in the size of the flexor tendons and the surrounding retinacular pulley system at the first annular pulley, which overlies the metacarpophalangeal joint. The flexor tendon catches when it attempts to glide through a relatively stenotic sheath, resulting in an inability to flex or extend the finger.

Epidemiology studies have suggested the phenomenon of concomitant CTS and trigger digits.^[1,2]^ A similar pathophysiological process underlying the 2 conditions is considered because of the findings of limited space in an enclosed anatomical region under both conditions. The clinical scenario of patients presenting with new-onset trigger digits after carpal tunnel release (CTR) raises the controversy of coincidence or complication. However, the relationship between CTS and trigger digits has been inconsistently reported across different studies.^[1,3–5]^ The current study analyzed nationwide population-based retrospective cohort data to assess the incidence of trigger digits after CTR.

## 2 Materials and methods

### 2.1 Data source

We conducted a retrospective cohort study with secondary data from the Longitudinal Health Insurance Database 2000 (LHID2000), a subset of the National Health Insurance Database (NHIRD) of Taiwan. The NHIRD, established in 1997, includes information on nearly 99% of the 23.74 million people in Taiwan and is managed and released by the National Health Research Institutes (NHRI) of Taiwan.^[6]^ The LHID2000 contains data of 1 million beneficiaries randomly selected from the year 2000 Registry for Beneficiaries of the NHIRD. These random samples (LHID2000) have been confirmed by the NHRI to be representative of Taiwanese residents. For each beneficiary, a unique identification number was used to link all insurance information and health care records. Diseases were defined on the basis of International Classification of Diseases, Ninth Revision, Clinical Modification (ICD-9-CM) codes. This study was approved by the Institutional Review Board of China Medical University (CMUH-104-REC2-115).

### 2.2 Sampled participants

The study patients were selected from among those in the LHID2000 diagnosed as having CTS (ICD-9-CM code 354.0) between 2000 and 2010. CTS patients aged 20 years and older who underwent CTR were classified as the CTR cohort, and patients with CTS who did not undergo CTR were classified as the non-CTR cohort. The date of the CTR was used as the index date. Patients with a history of trigger digits (ICD-9-CM code 727.03) before their index date and those younger than 20 years were excluded. For each CTR patient, 4 non-CTR controls were randomly selected from the remaining patients who had CTS but did not undergo CTR, frequency matched by the year of the index date, age (every 5-year span), and sex by using the same exclusion criteria. All patients were followed from the index date until the date of trigger digits diagnosis, withdrawal from the National Health Insurance program, or December 31, 2011.

### 2.3 Variables of interest and comorbidities

The demographic classifications of the CTR and non-CTR cohorts included sex, age (≤49, 50–64, and ≥65 years) and comorbidities. The baseline history of comorbidities in each patient was established for diabetes (ICD-9-CM code 250), hypothyroidism (ICD-9-CM code 244), wrist fracture (ICD-9-CM code 814), end-stage renal disease (ESRD; ICD-9-CM code 585), and rheumatoid arthritis (ICD-9-CM code 714).

### 2.4 Statistical analysis

The chi-squared test was used for categorical variables, and the Student *t* test was used for continuous variables. Cumulative incidences of trigger digits in the CTR and non-CTR cohorts were explored using the Kaplan–Meier method, and the differences were determined using log-rank tests. The incidence density rates of trigger digits (per 1000 person-year) were estimated according to sex, age, comorbidity, and follow-up period in both cohorts. Univariable and multivariable Cox proportional hazard regressions were used to compare the risk of developing CTR-associated trigger digits in the CTR and non-CTR cohorts. The variables used in the multivariable model were sex, age, and the comorbidities of diabetes, hypothyroidism, wrist fracture, ESRD, and rheumatoid arthritis. All statistical analyses were performed using SAS 9.4 software (SAS Institute, Cary, NC). We set the level of significance at a 2-sided *P* value of less than .05.

## 3 Results

**Figure 1:**
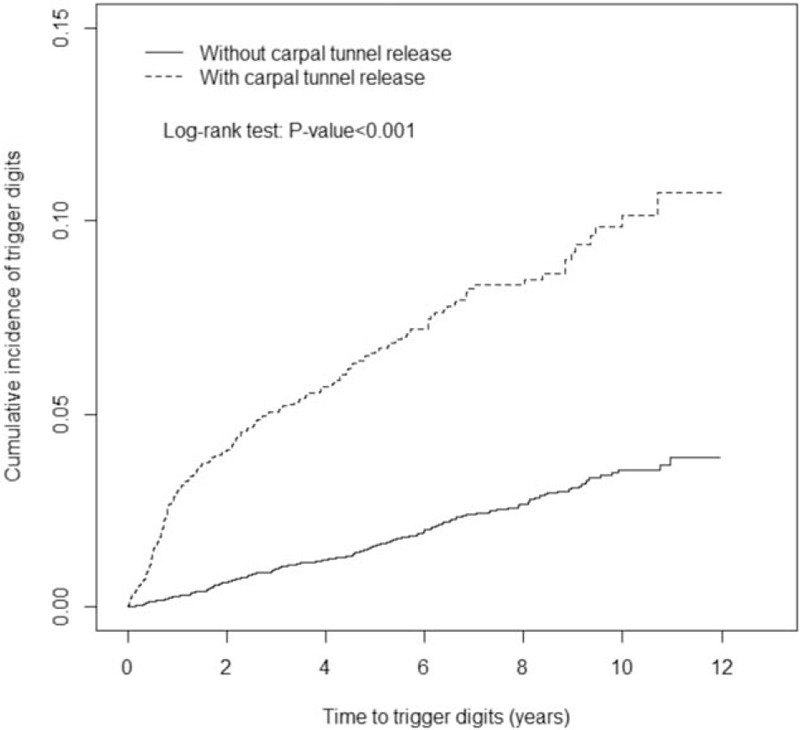
Cumulative incidence of trigger digits compared between carpal tunnel syndrome patients with and without carpal tunnel release using the Kaplan–Meier method.

We included 2605 patients in the CTR cohort and 10,420 patients in the non-CTR cohort. The age and sex distributions of the 2 cohorts were similar (Table 1). In both cohorts, 73.4% of the patients were women, 42.3% were aged ≤49 years, and the mean age was approximately 53 years. Diabetes, wrist fracture, and ESRD were more prevalent in the CTR cohort than in the non-CTR cohort (all *P* < .05). The mean follow-up periods until the development of trigger digits in the CTR and non-CTR cohorts were 5.58 and 5.90 years, respectively. The Kaplan–Meier survival analysis demonstrated that the cumulative incidence of trigger digits was 6.71% higher in the CTR cohort than in the non-CTR cohort (Fig. 1, *P* < .001). The overall incidence density rate of trigger digits was 3.70-fold higher in the CTR cohort than in the non-CTR cohort (12.6 vs 3.38 per 1000 person-year), with an adjusted hazard ratio (aHR) of 3.63 (95% confidence interval [CI] = 2.97–4.44) (Table 2). The sex-specific relative risks of developing trigger digits in the CTR cohort as compared with the non-CTR cohort were significantly higher for both women (aHR = 3.39, 95% CI = 2.74–4.21) and men (aHR = 6.01, 95% CI = 3.34–10.8). The age-specific aHR in the CTR patients as compared with non-CTR participants was higher for all age groups (aHR = 4.43, 95% CI = 3.18–6.17 for ≤49 years; aHR = 3.25, 95% CI = 2.47–4.26 for 50–64 years; and aHR = 3.12, 95% CI = 1.54–6.32 for ≥65 years). Among the patients without any comorbidities, the risk of trigger digits was 3.78-fold higher in the CTR cohort than in the non-CTR cohort (95% CI = 3.05–4.69). Furthermore, the analysis of HRs for developing trigger digits was stratified by follow-up periods (Table 3). Within 6 months of follow-up, the CTR cohort had a higher risk of trigger digits compared with the non-CTR cohort (aHR = 9.65, 95% CI = 5.27–17.7). Moreover, the risk of trigger digits in the CTR cohort was still significantly higher than that in the non-CTR cohort within 0.5 to 8 years of follow-up (aHR = 1.76, 95% CI = 1.07–2.90).

**Table 1:**
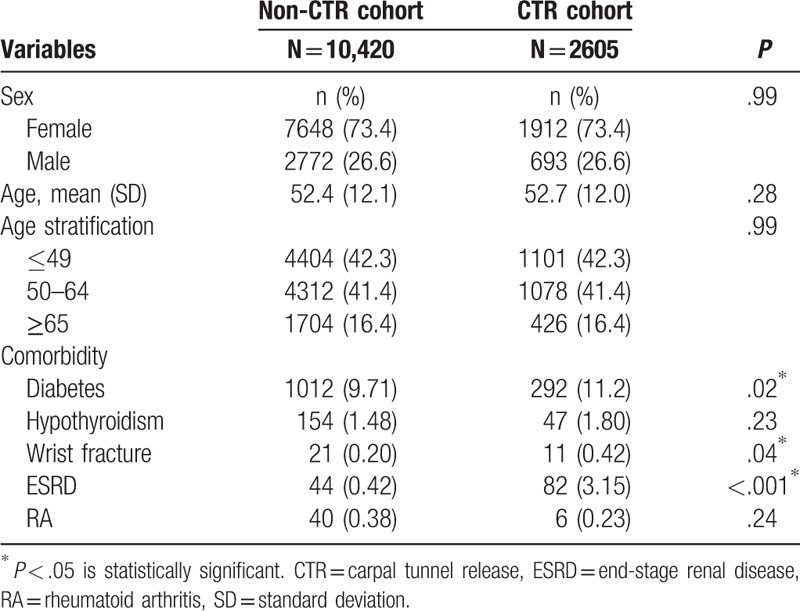
Demographic characteristics in carpal tunnel syndrome patients with and without CTR.

**Table 2:**
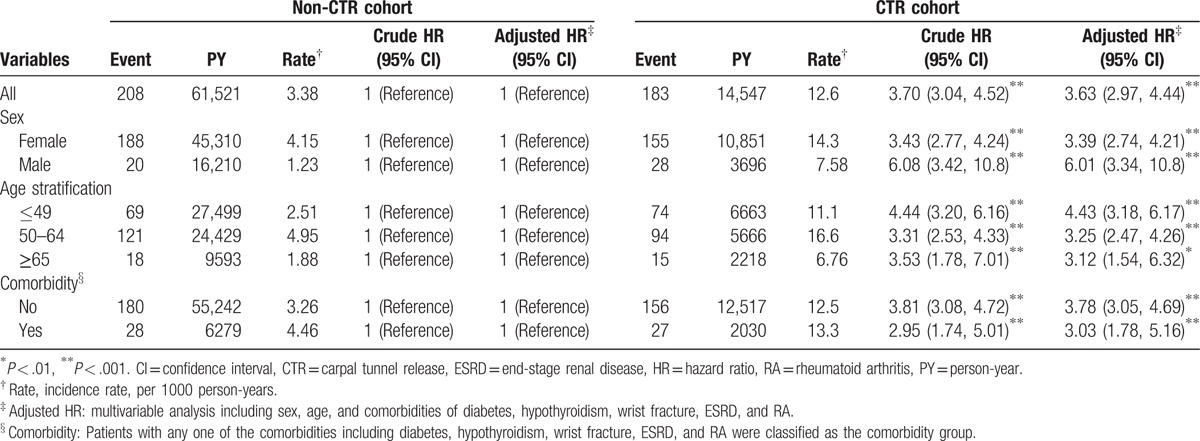
Comparison of incidence and hazard ratio of trigger digits stratified by sex, age, and comorbidity between carpal tunnel syndrome patients with and without carpal tunnel release.

**Table 3:**
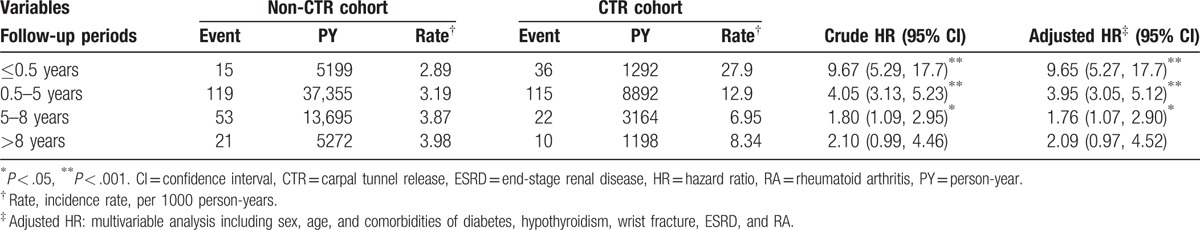
Trends of trigger digits risks by stratified follow-up years.

## 4 Discussion

According to previous systematic reviews, the incidence of trigger digits after CTR ranged from 0.4% to 31.7%, with most incidences developing within 6 months.^[7]^ Our findings demonstrate a significant difference in the incidence of trigger digits between the CTR and non-CTR cohorts. Patients with CTR had a 3.63-fold higher risk of developing trigger digits after surgery, compared with those without. This increased risk was consistent across different groups of sex and age. Moreover, the increased risk existed not only in all patients but also in an idiopathic subgroup of CTS patients who did not have any of the comorbidities mentioned. The risk of developing trigger digits was considerably elevated to 9.65-fold within 6 months after CTR and persisted until 8 years, but gradually decreased during the follow-up period.

Hombal and Owen^[8]^ presented the phenomenon that tenosynovial structures of varied degrees develop in the flexor digital sheaths after CTR. They considered that division of the flexor retinaculum contributed to the bowstring effect, which increased the friction force of the flexor tendons on proximal pulleys, resulting in the manifestation of new-onset trigger digits. The findings of our study highly suggest the possibility of increased opportunities for trigger digits development after CTR, which agrees with the hypothesis. We should include these findings in preoperative assessments for CTR.

Our CTR cohort contained a higher percentage of patients with ESRD compared with the control group. According to a survey in France, Tuppin et al^[9]^ reported that ESRD treated with dialysis was one of the comorbidities with the highest risk (relative risk = 3.3) associated with CTR compared with other conditions such as diabetes or hypothyroidism. The medical accessibility of patients with ESRD may contribute to their high surgical treatment rate.

An advantage of our study is that it is the first to examine the association between CTS, with and without releasing, and the incidence of trigger digits. In addition, a large sample was used to analyze trigger digits risk differences between CTS patients with and without CTR. Moreover, selection and nonresponse biases may have been minimized by the comprehensive coverage of the National Health Insurance system (>96% of the population).

The study has 2 limitations. First, we could not balance the severity of CTS between the 2 groups because the LHID2000 does not include information regarding symptoms and electrophysiological findings. The patients receiving CTR may be assumed to have more debilitating symptoms such as pain and paresthesia, or prominent neurological signs such as muscle atrophy. Second, the comorbidities in these 2 cohorts were not homogeneous, especially the percentages of ESRD, diabetes, and wrist fracture patients. Easy access to medical services in these 3 types of patients might explain their high operation rates for CTS.

## 5 Conclusion

CTR was significantly associated with the subsequent development of trigger digits, particularly within the first 6 postoperative months. We should integrate this information into comprehensive preoperative assessments of CTR, and inform patients of the possibility of trigger digits occurrence after surgery.

## Acknowledgments

The authors thank Taiwan Ministry of Health and Welfare Clinical Trial and Research Center of Excellence (MOHW105-TDU-B-212-133019), China Medical University Hospital, Academia Sinica Taiwan Biobank Stroke Biosignature Project (BM10501010037), NRPB Stroke Clinical Trial Consortium (MOST 104-2325-B-039-005), Tseng-Lien Lin Foundation, Taichung, Taiwan, Taiwan Brain DiseaseFoundation, Taipei, Taiwan, and Katsuzo and Kiyo Aoshima Memorial Funds, Japan for the support.

## References

[1] Rottgers SA Lewis D Wollstein RA Concomitant presentation of carpal tunnel syndrome and trigger finger. J Brachial Plex Peripher Nerve Inj 2009;25:13. 10.1186/1749-7221-4-13 PMC2743689 19706185

[2] Kumar P Chakrabarti I Idiopathic carpal tunnel syndrome and trigger finger: is there an association? J Hand Surg Eur Vol 2009;34:58–9. 18936127 10.1177/1753193408096015

[3] Harada K Nakashima H Teramoto K Trigger digits-associated carpal tunnel syndrome: relationship between carpal tunnel release and trigger digits. Hand Surg 2005;10:205–8. 16568515 10.1142/S0218810405002905

[4] Hayashi M Uchiyama S Toriumi H Carpal tunnel syndrome and development of trigger digit. J Clin Neurosci 2005;12:39–41. 15639409 10.1016/j.jocn.2004.08.005

[5] King BA Stern PJ Kiefhaber TR The incidence of trigger finger or de Quervain's tendinitis after carpal tunnel release. J Hand Surg Eur Vol 2013;38:82–3. 22791612 10.1177/1753193412453424

[6] Database NHIR. Taiwan, http://nhird.nhri.org.tw/en/index.html. (cited in 2015). Accessed December 21, 2015.

[7] Cheng HT Wu CI Hsu YC Coincidence or complication? A systematic review of trigger digits after carpal tunnel release. Plast Reconstr Surg 2015;136 (4 Suppl):21–2. 28686120 10.1080/2000656X.2017.1345751

[8] Hombal JW Owen R Carpal tunnel decompression and trigger digits. Hand 1970;2:192–6. 5520745 10.1016/0072-968x(70)90022-7

[9] Tuppin P Blotiere PO Weill A Carpal tunnel syndrome surgery in France in 2008: patients’ characteristics and management. Rev Neurol (Paris) 2011;167:905–15. 22035728 10.1016/j.neurol.2011.05.010

